# Trampoline-related fractures of the proximal tibia in children

**DOI:** 10.1186/s13018-021-02707-9

**Published:** 2021-09-08

**Authors:** Changhoon Jeong, Sang Uk Lee, Hyun Gyun Kim, Sun Young Joo

**Affiliations:** 1 Department of Orthopedic Surgery, Bucheon St. Mary’s Hospital, College of Medicine, The Catholic University of Korea, Incheon, Republic of Korea; 2 Department of Orthopedic Surgery, Incheon St. Mary’s Hospital, College of Medicine, The Catholic University of Korea, 56 Dong-su ro, Bupyeong-gu, Incheon, 21431 Republic of Korea

**Keywords:** Proximal tibia, Fracture, Trampoline, Children

## Abstract

**Background:**

Trampoline-related fractures of the proximal tibial metaphysis are common in children and have been linked to subsequent valgus deformity of the tibia. The purpose of this study was to investigate the characteristics of trampoline-related proximal tibial fractures in young children.

**Methods:**

We evaluated 40 patients with proximal tibial fracture after trampolining between 2013 and 2019. The median duration of follow-up was 18 months. Standing long leg radiographs were obtained at the last follow-up to evaluate angular deformity and limb length inequality in the patients. The measurements recorded include the lower limb length, mechanical lateral distal femoral angle (mLDFA), medial proximal tibial angle (MPTA), mechanical axis deviation (MAD), and anatomical tibio-femoral angle (aTFA). The anterior tilt angle (ATA) was measured using a lateral radiograph of the tibia.

**Results:**

The median age at injury was 40.0 months. Using trampoline with a heavier person was the most common mechanism of injury. aTFA and MAD were found to be increased towards the valgus at the last follow-up in our patient; however, the increase was not statistically significant (*p* = 0.692 and *p* = 0.973, respectively). The anterior tilt angle was increased in the injured leg at the last follow-up. But the change was not statistically significant (*p* = 0.09).

**Conclusions:**

Using trampoline with a heavier person carries the risk of trampoline-related proximal tibial fracture in young children. We did not find a significant change in limb alignment at a minimum of one year of follow-up.

## Background

As the recreational use of trampolines has increased drastically in the last 10 years [1–3], there are increasing concerns on trampoline injury worldwide. The National Electronic Injury Surveillance System in the USA shows that between 2002 and 2011, trampoline injuries resulted in nearly 100,000 emergency department visits each year and 29% of fractures [1]. Even though the American Academy of Pediatrics has warned against the use of trampolines by children under 6 years of age and has recommended that safety measures for trampoline use should include constant adult supervision, adequate protective padding, one jumper per trampoline, and the avoidance of flips and somersaults, a significant increase in the national incidence of pediatric trampoline-related fractures has been noted: from 35.3 per 100,000 person-years in 2008 to 53.0 per 100,000 person-years in 2017 [4, 5]. A similar tendency is noted in Korea. Trampoline-related injuries increased steadily as the trampoline parks and kids’ cafés increased [6]. The injuries increased steadily from 2011 to 2016, while the age at which injury occurred decreased gradually over the same period. Also, 2799 patients with trampoline injuries visited emergency departments during this period and fractures were sustained by 886 patients (31.7%), with the distal humerus (34%) and the proximal tibia (23%) being the most common fracture sites [6].

Proximal tibial metaphyseal fractures in children are among the most common trampoline-related injuries [7–9]. This fracture carries the risk of progressive valgus deformity of the tibia, even after fracture healing (Cozen’s phenomenon) [10–12]. Therefore, physicians are advised to inform parents of the possibility of progressing valgus deformity and the necessity of long-term follow-up. This could be a burden not only on the physicians, but also on the care giver as well. Furthermore, if the injury occurs in a commercial park, it may cause legal issues, too.

Contrary to previous reports on Cozen’s phenomenon, recent studies show that trampoline-related proximal tibial metaphyseal fractures may not progress into valgus deformity [13, 14]. Boyer et al. reported seven children with trampoline-related proximal tibial metaphyseal fracture, none of which progressed into valgus deformity [14]. However, the median follow-up period was not reported, and the result was based on the clinical observation of the referring physicians, without radiologic confirmation.

In this study, we aimed to investigate the characteristics of trampoline-related proximal tibial fractures in young children and to evaluate the changes in limb alignment after healing.

## Methods

**Fig. 1:**
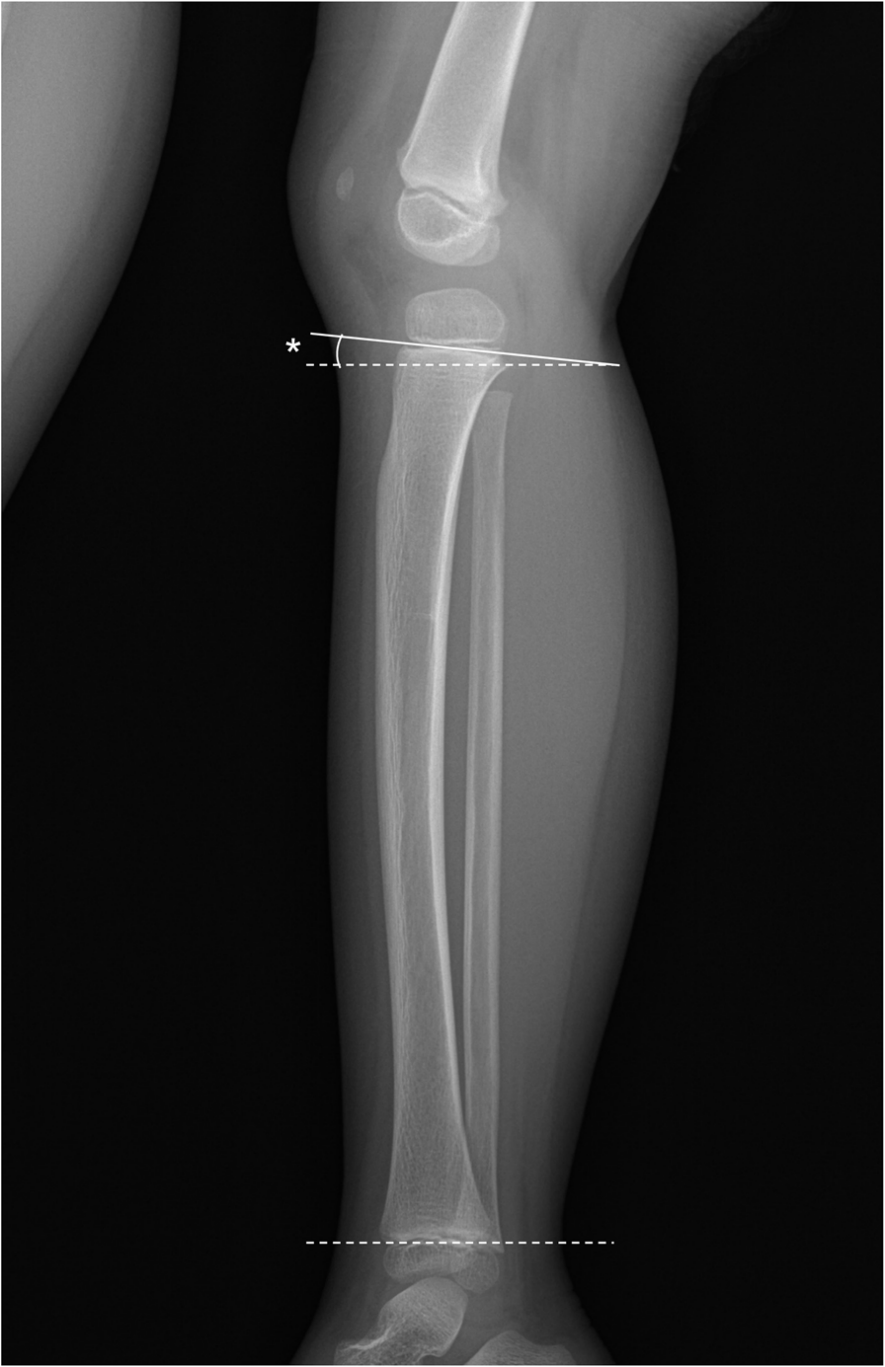
The anterior tilt angle. The proximal line of the angle is defined by drawing a tangent between the dorsal and mid-point of the physis. The distal line of the angle is defined by drawing a tangent between the dorsal and ventral epiphyseal plate of the distal physis

This retrospective study was performed after approval from our Institutional Review Board. We included patients who visited our institution between 2013 and 2019 after trampoline injury of the proximal tibia and who were able to report for follow-up till at least 12 months after injury. The exclusion criteria were as follows: lost to follow-up, congenital or acquired deformity of either the injured or the contralateral uninjured lower limb, or an unknown mechanism of injury. Finally, 40 patients were selected. Patient demographics, such as age, sex, date of presentation, and the duration of follow-up, were collected from the medical records. The mechanism of injury and the side of injury were also recorded. Anterior-posterior and lateral radiographs of the knee revealed a fracture pattern, which was categorized as cortical buckling, anterior scooping of the notch for the tibial tubercle, complete cortical break, and fracture obliquity directed toward the physis. Coexisting fibular fracture was also observed (Fig. 1).

**Fig. 2:**
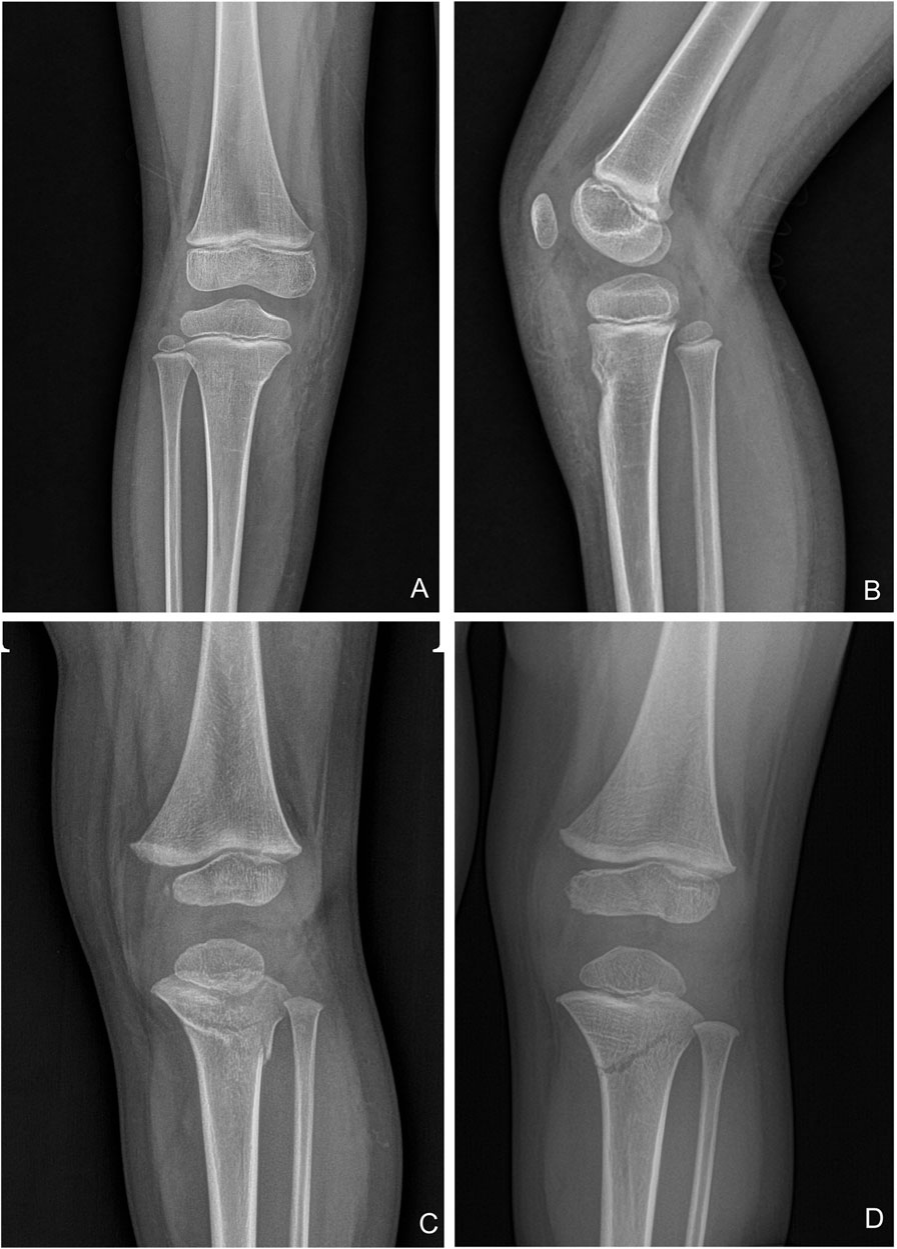
Fracture pattern. Cortical buckling (**A**), anterior scooping of the notch for the tibial tubercle (**B**), complete cortical break (**C**), and fracture obliquity directed toward the physis (**D**)

Standing long leg radiographs were obtained at the last follow-up to evaluate angular deformity and limb length inequality in the patients. The measurements recorded included the lower limb length, mechanical lateral distal femoral angle (mLDFA), medial proximal tibial angle (MPTA), mechanical axis deviation (MAD), and anatomical tibio-femoral angle (aTFA). The anterior tilt angle (ATA) was measured on a lateral radiograph of the tibia that included the proximal and distal tibial epiphyseal plate (Fig. 2). The proximal line of the angle was defined by drawing a tangent between the dorsal and mid-point of the physis (the anterior point of the physis may be used if the line intersects the mid-point). The distal line of the angle was defined by drawing a tangent between the dorsal and ventral epiphyseal plate of the distal physis [15]. Patients were treated with long leg cast immobilization for 3-4 weeks, according to their age. None of the patients underwent surgery for fracture reduction. Radiographic measurements of the injured leg at final follow-up were compared with those of the uninjured leg. Statistical analyses were performed using SPSS software (version 21; IBM Co., Armonk, NY, USA). Normal distribution was evaluated using the Kolmogorov-Smirnov test, Student *t* test, and Mann-Whitney *U* test and was utilized for statistical analysis. Statistical differences were considered when the *p* value was less than 0.05.

## Results

Fifteen boys (37.5%) and 25 girls (62.5%), with a median age of 40.0 months (interquartile range: 29.0 to 52.0 months) at the time of injury were included in this study. The right side was involved in 21 patients (52.5%) and the left side in 19 patients (47.5%). With a median follow-up period of 18.0 months (interquartile range: 12.0 to 25.0 months), the median age at last follow-up was 63.0 months (interquartile range: 48.0 to 70.0 months).

Jumping on the trampoline with a heavier person was the most common mechanism of injury. No patient had collided with another person. Thirty patients (76.9%) had been injured by jumping on the trampoline with heavier kids, while nine (23.1%) were injured while jumping on the trampoline with one of their parents. The place of injury for all cases was the commercial park, at a “kid’s café.” None of the patients were injured on trampolines at home. Eight of the 40 patients (20.0%) were initially misdiagnosed as sprain or contusion of the leg at another hospital (Table 1).

**Table 1:** Patient’s demographics

	Boys (*N*=15)	Girls (*N*=25)	Total (*N*=40)
Age at injury (mo)	44.0 (34.0–54.0)	37.5 (25.5–48.8)	40.0(29.0–52.0)
Site of injury			
Rt	9 (60.0%)	12 (48.0%)	21 (52.5%)
Lt	6 (40.0%)	13 (52.0%)	19 (47.5%)
Duration of follow-up (mo)	17.0 (13.0–25.0)	19.0 (12.0–25.0)	18.0 (12.0–25.0)
Age at last follow-up (mo)	65.0 (51.0–73.0)	61.0 (38.8–68.8)	63.0 (48.0–70.0)
Initial misdiagnosis	2 (5.0%)	6 (15.0%)	8 (20.0%)

Data presented as median (interquartile range) or *n* (%)

Anterior scooping of the notch for the tibial tubercle was the most common fracture pattern, seen in 19 patients (48.7%), followed by buckling of the cortex in 18 patients (46.2%). None of the patients had a concomitant fibular fracture (Table 2). The mean mLDFA of the injured leg at the final follow-up was 89.3 ± 2.1° (range: 85.6 to 93.7°) and that of the uninjured leg was 88.9 ± 1.8° (range: 86.1 to 94.3°); the difference between the two groups was not statistically significant (*p* = 0.450). The mean MPTA of the injured leg at the last follow-up was 89.2 ± 1.6° (range: 86.0 to 92.9°) and that of the uninjured leg was 88.9 ± 1.9° (range: 86.1 to 93.2°); this difference was also not significantly different (*p* = 0.510). The difference in the aTFA between the injured and uninjured legs was also statistically insignificant (*p* = 0.692). The mean limb length discrepancy was 1.0 ± 2.8 mm (range: − 3.6 to 7.1 mm). The injured limb was longer than the contralateral limb in 25 patients (64.1%), with an average limb-length discrepancy of 2.7 ± 1.9 mm (range: 0.38 to 7.13 mm). The mean MAD of the injured leg was 0.6 ± 5.0 mm (range: − 14.6 to 14.8 mm) and the mean MAD of the uninjured leg was 0.6 ± 4.4 mm (range: − 14.6 to 11.1 mm). The MAD and limb length discrepancy were statistically insignificant, with *p* values of 0.973 and 0.938, respectively. The mean ATA in the injured leg was increased, with an average of 2.2 ± 4.3° (range: − 6.1 to 9.1°), compared to − 0.8 ± 3.5° (range: − 8.4 to 6.7°) on the uninjured side, indicating a compression of the fracture site. However, the difference was not statistically significant (*p*=0.099; Table 3).

**Table 2:** Fracture pattern

Fracture pattern	*N*	%
Buckling of the cortex	18	46.2
Anterior tibial increased scoop	19	48.7
Complete cortical break	8	20.5
Oblique extension toward physis	9	23.1
Fibular fracture	0	0

**Table 3:** Comparison of radiographic parameters

Variable	Injured leg	Uninjured leg	*p*-value
aTFA (°)	6.3 ± 3.2	6.0 ± 2.8	0.692
mLDFA (°)	89.3 ± 2.1	88.9 ± 1.8	0.450
MPTA (°)	89.2 ± 1.6	88.9 ± 1.9	0.510
MAD (mm)	0.6 ± 5.0	0.6 ± 4.4	0.973
ATA (°)	2.2 ± 4.3	-0.8 ± 3.5	0.099*
Limb length (mm)	465.4 ± 64.9	464.3 ± 64.1	0.938

Data presented as mean ± standard deviation

*aTFA* anatomical tibio-femoral angle, *mLDFA* mechanical lateral distal femoral angle, *MPTA* medial proximal tibial angle, *MAD* mechanical axis deviation (+ means valgus deviation), *ATA* anterior tilt angle

*Tested by Mann Whitney *U* test

## Discussion

It has been over 50 years since Dr. Lewis Cozen first reported progressive valgus deformity after proximal tibial metaphyseal fracture. However, the prevalence and etiology of Cozen’s phenomenon are still not fully understood. The prevalence of Cozen’s phenomenon after proximal tibial metaphyseal fractures has been reported to be up to 90%, [16–18] although recent studies show a lower prevalence [17]. The suggested mechanisms of valgus deformity after proximal tibial fractures include asymmetric activity of the medial portion of the proximal tibial physis (overgrowth), tethering effect of the fibula, inadequate reduction, interposed soft tissue (pes anserinus), and loss of the tethering effect of the pes anserinus. Early weight-bearing produced developmental valgus and physeal arrest of the lateral aspect of the proximal tibial physis [19].

Our retrospective study investigated the characteristics of trampoline-related proximal tibial fractures in young children, including Cozen’s phenomenon. Based on our study, the characteristics of the trampoline-related proximal tibial fracture is that this fracture occurred in younger children. We found that the fractures are easy to misdiagnose as sprain or contusion, occur mostly by jumping on the trampoline with a heavier person, and do not cause progressive valgus deformity. The median age of our patients was 40.0 months (interquartile range: 29.0 to 52.0 months), younger than the entire patients’ group of trampoline-related injury in the Korean national database, which was 5.4 years [6]. The children with proximal tibial fractures who suffered from trampoline-related injuries in our study were younger than those in reports from the United States and Europe [6]. This is important because the mechanism of injury of proximal tibial fractures during trampolining is different in younger children than that seen in older children, and this difference accounts for the occurrence of Cozen’ phenomenon in the younger children. Older children fell off the trampoline, got injured by or collided with another person or structure; therefore, valgus force could have been present [20]. Jumping with a heavier person was the most common mechanism of injury. However, none of our patients got injured by a collision or fell off the trampoline; they usually got injured while trampolining with heavier person without collision. Our findings are well supported by a report by Boyer et al [13].

Several studies have revealed that approximately three-quarters of injuries occurred when multiple people were using the trampoline simultaneously [4]. The smallest participants were up to 14 times more likely to sustain injury relative to their heavier playmates, who could create more recoil of the mat and springs and greater upward impaction forces [3]. These forces must be absorbed by the falling body and can be larger than when landing on solid ground. The American Academy of Pediatrics warns that the safety measures for trampoline use include constant adult supervision and one jumper per trampoline. In the United States, backyard trampolines are common [21]. However, trampoline activity in Korea has unique environmental differences. Due to space limitations, backyard or home trampolines are not popular in Korea. Rather, trampolines have been installed in small indoor playgrounds, called “kids’ cafes,” and small playrooms in restaurants. Therefore, there is an increased risk of multiple simultaneous jumpers.

Nine of the 40 patients in our study (23.1%) were injured while jumping on the trampoline with one of their parents. This may be because of lack of awareness that jumping with their parents could also be dangerous. Therefore, parent education is necessary to prevent trampoline-related injury in young children.

Initial misdiagnosis as a sprain or contusion of the lower leg was common, encountered in 8 patients (20.0%) in our study. This could be due to the non-contact mechanism of injury, because of which some children were able to walk. Also, young children are uncooperative on physical examination, making it difficult to localize the source of pain. Furthermore, initial radiographic changes, such as anterior scooping of the notch for the tibial tubercle or buckling of the cortex, are usually subtle on plain radiographs. Therefore, without understanding the mechanism of injury, a misdiagnosis of sprain or contusion is possible. Magnetic resonance imaging (MRI) or ultrasonography has been suggested for diagnosing trampoline injury in children with normal radiographs [22]. Although MRI is an excellent tool for identifying occult fractures, young children are unable to lie still, and sometimes sedation under general anesthesia may be necessary to obtain an adequate MRI examination. Ultrasonography could also be useful in the diagnosis of occult fractures [23]. Simanovsky et al. prospectively evaluated 58 children with an acute ankle and wrist injury who were suspected of having a fracture despite normal radiographs; 15 patients with positive ultrasonographic findings were diagnosed with a fracture on follow-up radiographs. The advantages of ultrasound imaging include bedside availability and the relative ease of performing repeated examinations. Furthermore, imaging is real-time and free of harmful radiation. However, it is highly operator-dependent.

Several mechanisms for the trampoline-related fracture of the proximal tibial metaphysis have been suggested. They include a fall or incorrect landing, collision with other jumpers, falling off the trampoline, or contact with other structures. Mubarak et al. classified 135 pediatric proximal tibial fractures into four groups, according to the direction of force of injury [24]. Of the 135 fractures, 28 (20.7%) were classified into the valgus group; the prototypical activity of this group was jumping on the trampoline, wherein force was applied to the lateral aspect of the extended knee, producing the greenstick fracture of the proximal tibial metaphysis. In a study by Kim et al, of 43 patients, 19 (44.2%) showed varus angulation more than 2° compared to the uninjured leg, 20 remained neutral, and only 4 patients showed valgus angulation [8]. They, thus, concluded that the varus force was more common than the valgus force. Compression force by the recoiling mat was suggested by Boyer et al. When a small child lands on the upward moving mat at the time when its elasticity is reversed by recoil and the springs are shortening to their unstretched length, significant upward impaction force is applied to the descending child’s legs. Therefore, it is possible to get injured without direct impact with other structures or collision with other jumpers. Our results support the findings of Boyer et al.’s study [13]. Valgus deformity was not clinically significant in our study. aTFA and MAD were increased towards valgus at the last follow-up in our patients. However, the increase was not statistically significant (*p* = 0.692 and *p* = 0.973, respectively). The ATA was increased in the injured leg at the last follow-up, suggesting that compression force had been applied. However, even this difference was statistically insignificant (*p* = 0.099). We think that in young children who are using trampoline with a heavier person, the prototypic injury is compression force created by the recoiling mat. Additional varus or valgus force may be present, according to the position of the patient.

None of our patients showed Cozen’s phenomenon during the follow-up period. This could be because unlike other proximal metaphyseal fractures that cause Cozen’s phenomenon, our patients had non-displaced and non-angulated linear or buckle fractures. Therefore, relatively mild impact was applied to the proximal tibia, not enough to cause subsequent valgus deformity. This unique mechanism of injury probably did not cause much disruption of the epiphyseal-metaphyseal region. Absence of fibular fracture may also play a role in preventing Cozen’s phenomenon. Although it is still controversial whether or not the intact fibula is a risk factor for Cozen’s phenomenon, a recent study shows an increased risk of progress into valgus deformity with a concurrent fibular fracture [17, 25].

Our study had several limitations. Firstly, although we excluded patients in whom the mechanism of injury was unknown, recall bias may have existed. Fortunately, in some patients, we could observe the mechanism by viewing recordings from the closed-circuit television installed around the trampoline. Secondly, since our patients had no concomitant fibular fracture, the role of fibular fracture could not be assessed.

## Conclusion

Using trampoline with a heavier person carries the risk of proximal tibial fracture in young children. Radiologic findings may be subtle and anterior compression was observed to be the most common fracture pattern. Initial misdiagnosis as sprain or contusion is common. Therefore, physicians should be aware of a possible proximal tibial fracture when a child presents with limping or refuses to walk after jumping on the trampoline with a heavier person. These fractures are relatively benign, changes in limb alignment after healing were not significant with a minimum one year of follow-up.

## Data Availability

Not applicable

## Abbreviations

mLDFA: Mechanical lateral distal femoral angle
MPTA: Medial proximal tibial angle
MAD: Mechanical axis deviation
aTFA: Anatomical tibio-femoral angle
ATA: Anterior tilt angle
MRI: Magnetic resonance imaging
